# Immunogenicity and Tolerance of BNT162b2 mRNA Vaccine in Allogeneic Hematopoietic Stem Cell Transplant Patients

**DOI:** 10.3390/vaccines12020174

**Published:** 2024-02-08

**Authors:** Ahmed Amine Ben Khlil, Imen Zamali, Dorra Belloumi, Mariem Gdoura, Ghassen Kharroubi, Soumaya Marzouki, Rym Dachraoui, Insaf Ben Yaiche, Soumaya Bchiri, Walid Hamdi, Manel Gharbi, Ahlem Ben Hmid, Samar Samoud, Yousr Galai, Lamia Torjmane, Saloua Ladeb, Jihene Bettaieb, Henda Triki, Nour Ben Abdeljelil, Tarek Ben Othman, Melika Ben Ahmed

**Affiliations:** 1 Department of Clinical Immunology, Institut Pasteur de Tunis, Tunis 1002, Tunisia; ahmedamine.benkhlil@etudiant-fmt.utm.tn (A.A.B.K.); imen.zamali@fmt.utm.tn (I.Z.); walid.hamdi@pasteur.tn (W.H.); ahlem.benhmid@pasteur.tn (A.B.H.); samar.samoud@pasteur.tn (S.S.); yousr.galai@pasteur.tn (Y.G.); 2 Faculté de Médecine de Tunis, Université Tunis El Manar, Tunis 1068, Tunisia; dorra.belloumi@fmt.utm.tn (D.B.); ghassen.kharroubi@pasteur.tn (G.K.); rim.dachraoui@etudiant-fmt.utm.tn (R.D.); insaf.benyaiche@fmt.utm.tn (I.B.Y.); lamia.torjemane@fmt.utm.tn (L.T.); saloua.ladeb@fmt.utm.tn (S.L.); jihene.bettaieb@pasteur.tn (J.B.); henda.triki@pasteur.tn (H.T.); nour.benabdeljelil@fmt.utm.tn (N.B.A.); tarek.benothman@fmt.utm.tn (T.B.O.); 3 Laboratory of Transmission, Control and Immunobiology of Infections (LR16IPT02), Institut Pasteur de Tunis, Tunis 1002, Tunisia; soumaya.marzouki@pasteur.tn (S.M.); soumaya.bchiri@fst.utm.tn (S.B.); 4 Department of Hematology and Transplant, Centre National de Greffe de Moelle Osseuse, Tunis 1006, Tunisia; 5 Laboratory of Virology, Institut Pasteur de Tunis, Tunis 1002, Tunisia; mariem.gdoura@pasteur.tn (M.G.); gmanel599@gmail.com (M.G.); 6 Faculty of Pharmacy, University of Monastir, Monastir 5000, Tunisia; 7 Department of Medical Epidemiology, Institut Pasteur de Tunis, Tunis 1002, Tunisia

**Keywords:** COVID-19, vaccines, humoral immunity, cellular immunity

## Abstract

Background: Allogeneic hematopoietic stem cell transplantation (ASCT) induces acquired immunodeficiency, potentially altering vaccine response. Herein, we aimed to explore the clinical tolerance and the humoral and cellular immune responses following anti-SARS-CoV-2 vaccination in ASCT recipients. Methods: A prospective, non-randomized, controlled study that involved 43 ASCT subjects and 31 healthy controls. Humoral response was investigated using the Elecsys^®^ test anti-SARS-CoV-2. Cellular response was assessed using the QFN^®^ SARS-CoV-2 test. The lymphocyte cytokine profile was tested using the LEGENDplex™ HU Th Cytokine Panel Kit (12-plex). Results: Adverse effects (AE) were observed in 69% of patients, encompassing pain at the injection site, fever, asthenia, or headaches. Controls presented more side effects like pain in the injection site and asthenia with no difference in the overall AE frequency. Both groups exhibited robust humoral and cellular responses. Only the vaccine transplant delay impacted the humoral response alongside a previous SARS-CoV-2 infection. Noteworthily, controls displayed a Th1 cytokine profile, while patients showed a mixed Th1/Th2 profile. Conclusions: Pfizer-BioNTech^®^ anti-SARS-CoV-2 vaccination is well tolerated in ASCT patients, inducing robust humoral and cellular responses. Further exploration is warranted to understand the impact of a mixed cytokine profile in ASCT patients.

## 1. Introduction

COVID-19, caused by SARS-CoV-2, profoundly disrupted the healthcare system during the pandemic that started in March 2020 [1]. In addition to the saturation of acute medicine services (emergency care, intensive care units, etc.), non-COVID-19 hospital activity has been slowed down to protect patients from the risk of contamination. This is particularly true for services caring for immunocompromised patients [2]. Massive vaccination was the only way to control the pandemic and reduce direct and indirect morbidity and mortality. Anti-SARS-CoV-2 vaccines with proven efficacy have been developed and were capable of reducing the individual and collective effects of COVID-19. However, limited data were available on the safety and efficacy of these vaccines in immunocompromised patients, who are often excluded from phase III trials.

The vaccine response of allogeneic hematopoietic stem cell transplant (ASCT) patients would probably be of poorer quality and of shorter duration compared to immunocompetent subjects. The underlying disease, the immunosuppressive treatment (in particular anti-CD20), and the graft versus host reaction or GVHD (Graft Versus Host Disease) could indeed inhibit the post-vaccination immune response. This has been demonstrated with classic influenza [3] or pneumococcal [4] vaccines. However, highly immunogenic vaccines have been proven to be effective in this population, in particular by maintaining a minimum period of 3 months post-transplant before getting vaccinated [5]. The scientific societies have established recommendations for organizing anti-SARS-CoV-2 vaccination in ASCT recipients with periodic updates. The only exclusion criteria being a delay of less than 3 months from the allograft and the presence of an uncontrolled acute or chronic GVHD. Prioritized vaccination was administered to patients who had received an allograft in the last 3 months to 3 years, patients over 3 years old that were receiving systemic immunosuppressants and patients aged over 50 with comorbidities.

The preferred use of mRNA vaccines has been advocated by the Francophone Society for Bone Marrow Transplantation and Cellular Therapy (SFGM-TC) and the European Society for Blood and Bone Marrow Transplantation (EBMT). The SFGM-TC, in its latest recommendations [6] published in September 2021, recommended a third vaccine dose for HSC transplant recipients in the anti-SARS-CoV2 primary vaccination scheme. However, given the possibility of developing an optimal serological response from the second dose, the third dose was left to the caution of the transplant team in charge of the patient. Booster vaccination with an mRNA vaccine spaced from the primary vaccination (i.e., at 6 months) was recommended in immunocompromised patients, including ASCT patients. Therefore, it was important to assess the response after primary vaccination in ASCT recipients and its kinetics, in order to judge the usefulness of a third and booster doses. In fact, conflicting results were reported about the quality of the anti-SARS-CoV2 vaccine response in ASCT patients compared to the general population as showcased in a recent meta-analysis [7]. Moreover, few studies have assessed both humoral and cellular responses to COVID vaccines. Herein, we aimed to evaluate the humoral and cellular responses to BNT162b2 mRNA vaccine in ASCT patients in comparison to a healthy group and to identify factors associated with a putative poor vaccine response. Moreover, tolerance to such vaccine in ASCT patients has also been monitored.

## 2. Materials and Methods

### 2.1. Ethic Statement

The study was approved by the ethic committee of Pasteur Institute of Tunis (2021/17/I/V1). All patients provided written informed consent for the collection of samples and the subsequent analysis. All research was conducted according to the declaration of Helsinki principles.

### 2.2. Study Population

This was a prospective non-randomized controlled study, carried out between September 2021 and September 2022 at the Hematology and Transplantation Department of the National Center for Bone Marrow Transplantation of Tunis (CNGMO) (Tunis, Tunisia) in collaboration with the Department of Clinical Immunology at Pasteur Institute of Tunis (Tunis, Tunisia).

Patients undergoing (ASCT at the CNGMO between 2017 and 2021) were considered for this study under the following criteria:

- Eligibility for vaccination: aged over 15 years and at least 3 months post-ASCT without uncontrolled GVHD*. *Chronic GVHD is considered active when there is an evolving symptomatology that varies depending on the affected organ (for example, wheezing dyspnea in pulmonary GVHD, cytolytic and cholestatic hepatopathy in hepatic GVHD, etc.). It is classified as uncontrolled if clinical and paraclinical manifestations do not improve or worsen despite an optimal immunosuppressive treatment.
- Having received two doses of anti-SARS-CoV-2 BNT162b2 mRNA vaccine.
- Providing their written, free, and informed consent to participate in the study.

Eligible patients who have received a single dose of vaccine in the presence of a declared history of SARS-CoV-2 infection were excluded.

Patients were recruited during routine consultations or through telephone interviews. A certificate indicating priority vaccination was provided to all patients, either by hand or sent by email. Once the vaccination date had been set, the investigating physician was notified to monitor clinical tolerance and to set the sampling date for the evaluation of the immune response. Clinico-biological data related to the patient, the underlying disease, and the transplantation were collected. All patients were invited for immunological follow-up of the vaccine response at 6 months after the second dose of the vaccination.

The controls were randomly selected, via the evax.tn App database (https://evax.tn, accessed on 29 July 2021), the national Tunisian Platform for monitoring COVID-19 vaccination, from all citizens over the age of 15, vaccinated in the governorate of Tunis with BNT162b2 mRNA vaccine over the same period as the patients included. Those who consented to participate in the study were included in the control group.

### 2.3. Sampling

The peripheral blood samples were collected 2 to 3 weeks after the second dose of the vaccine. Five milliliters of whole blood were collected in a tube without anticoagulant for the serology. One milliliter of whole blood was collected in each of the 4 heparinized whole blood tubes dedicated to the cellular study (Qiagen^®^, Hilden, Germany).

### 2.4. Peripheral Anti-N and Anti-S Antibodies Measurement

Total anti-S-RBD (Receptor-Binding Domain) antibodies were quantified using the commercial test, Elecsys^®^ Anti-SARS-CoV-2 S (Cat number: 09203095190, Roche^®^ Diagnostic, Basel, Switzerland), on the Cobas^®^ e411 analyzer. This quantitative test is calibrated against the first WHO international standard 20/136 from the National Institute for Biological Standards and Control, UK. The obtained results are expressed in U/mL, which is equivalent to Binding Antibody Unit per mL. The sensitivity cut-off is equal to 0.80 U/mL, indicating a previous contact with the virus, and the antibody cut-off, equal to 15.0 U/mL, indicates the presence of neutralizing antibodies with a positive predictive value of 100% according to the manufacturer. All sera were first analyzed without dilution. When the test indicated a result higher than the upper limit of quantification, which is 250 U/mL, the sera was diluted at 1/200, and the precise level was obtained after multiplying by the dilution factor. However, when diluted sera are still quantified to be higher than 250 U/mL, the result is retained as 5000 U/mL.

Sera were also tested for the detection of the total anti-N specific antibodies by the commercial test Elecsys^®^ anti-SARS-CoV2 qualitative assay (Cat number: 09289267119, Roche^®^ Diagnostic, Rotkreuz, Switzerland) on the Cobas^®^ e411 analyzer. This test is a qualitative assay with results expressed as an index (reference value of <1.0).

### 2.5. Cellular Immunity Analyses

The CD4^+^ and CD8^+^ T cell responses were evaluated using the Quantiferon SARS-CoV-2 (Qiagen). This assay consists of four antigen tubes, Nil, Mitogen, SARS-CoV-2 Ag1, and SARS-CoV-2 Ag2. Nil and Mitogen BCTs are intended to be used as negative and positive controls, respectively. SARS-CoV-2 Ag1 and Ag2 use a combination of antigen peptides specific to SARS-CoV-2 to stimulate lymphocytes involved in cell-mediated immunity in heparinized whole blood. The QFN SARS CoV-2 Ag1 tube contains CD4+ epitopes derived from the S1 subunit (Receptor-Binding Domain) of the Spike protein, and the Ag2 tube contains CD4+ and CD8+ epitopes from the S1 and S2 subunits of the Spike protein. Samples were processed according to manufacturer’s guidelines. IFN-γ concentration in IU/mL was then measured in the plasma from the stimulated samples using enzyme-linked immunosorbent assay (ELISA). The value was calculated by subtracting the Nil value (representing the baseline IFN-γ production for each patient) from the SARS-CoV-2 Ag1 and Ag2 values. Positive response was defined as a value of >0.15 IU/mL.

Next, the concentration of 12 cytokines (IL-2, IL-4, IL-5, IL-6, IL-9, IL-10, IL-13, IL-17A, IL-17F, IL-22, IFN-γ, and TNF-α) in the plasma of unstimulated and stimulated samples from 10 patients and 10 controls was quantified with multiplex technique using the “LEGENDplex™ HU Th Cytokine Panel (12-plex) of Biolegend^®^ (Th1-Th2-Th17), according to manufacturer procedures. Briefly, the capture antibody-conjugated beads were first incubated with plasma or standard controls for 60 min, then with biotinylated detection antibodies for 30 min, and finally with streptavidin-PE for 20 min. The fluorescence signals of the beads were acquired using a flow cytometer (FACS Canto II, Becton Dickinson).

### 2.6. Statistical Analysis

Data were analyzed using the SPSS version 25 software. For quantitative variables, we calculated medians and extreme values. For the qualitative variables, the percentages of positive and negative individuals in each group were calculated. The comparison of the quantitative variables was performed using the Student’s *t* test if the variable follows a normal distribution, otherwise the non-parametric tests (e.g., Mann–Whitney *U* test) were used. The comparison of the qualitative variables was carried out using the Chi-square test or the Fisher’s exact test with a *p* of less than 0.05 considered as significant. As in all statistical analyses, an alpha error risk of 5% was chosen. Univariate analyses were carried out to study the factors associated with the response. The variables used in the multivariate analysis are those for which the value of “*p*” was less than 0.2 in the univariate analysis.

## 3. Results

### 3.1. Study Population Description

**Figure 1:**
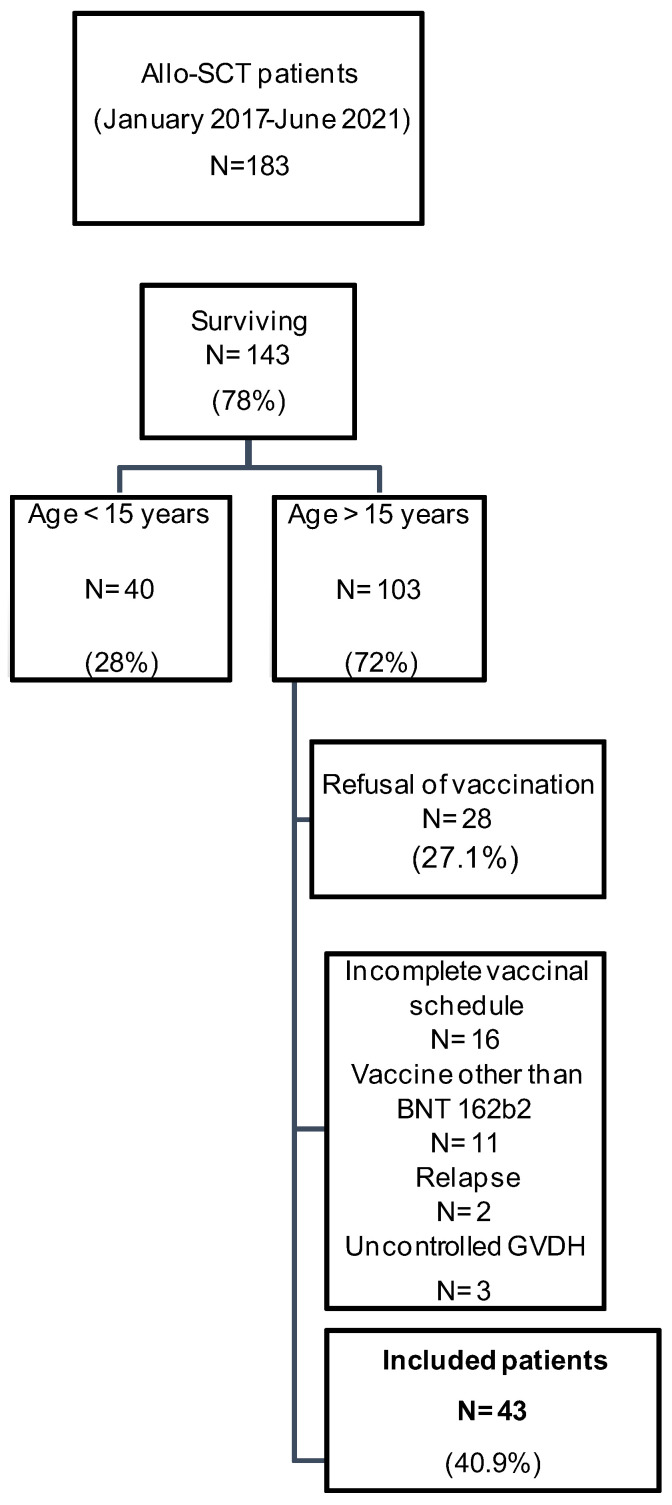
Study design. The study included all living ASCT patients aged 15 or more, fully vaccinated against SARS-CoV-2 with the Pfizer-BioNtech vaccine, and who were not in relapse of their underlying disease or had an uncontrolled GVHD (*n* = 43).

Between January 2017 and June 2021, 183 patients underwent ASCT as a part of the treatment of aplastic anemia or hematological malignancies in the Department of Hematology and Transplant of the CNGMO (Tunis, Tunisia). Among these patients, 43 met the inclusion criteria of the present study (Figure 1). The median age was 31 years (17–47). The sex ratio was 1.26 (24 men versus 19 women). Patients’ medical history included diabetes (*n* = 2), hypothyroidism (*n* = 2), asthma (*n* = 2), hepatitis B (*n* = 2), and pulmonary tuberculosis (*n* = 1). Four patients had a previous symptomatic COVID-19 infection. The diagnosis of this infection was confirmed using reverse transcriptase-polymerase chain reaction (RT-PCR) in three patients and by a suggestive chest computed tomography with negative RT-PCR in one patient. The presentation was mild in three patients and moderate in one patient. The evolution was favorable for all patients. The median duration between the previous infection and vaccination was 4 months with extremes ranging from 3 to 7. The hemopathies that indicated ASCT were a malignant blood disease (acute leukemia, myelodysplastic syndrome, Hodgkin’s lymphoma, and chronic myeloid leukemia) in 86% of cases (*n* = 37) and aplastic anemia in 14% of cases (*n* = 6). The source of the graft was peripheral stem cells in 49% of cases (*n* = 21) and bone marrow in 51% of cases (*n* = 22). ASCT was complicated by acute GVHD in 15 patients (35%), indicating corticosteroid therapy with a good evolution under treatment. The main localization was cutaneous involvement followed by digestive involvement. Controlled chronic GVHD was noted in 21 patients (48.8%). The median duration between ASCT and vaccination was 2 years and 4 months with extremes ranging from 3 months to 4.5 years. At the time of vaccination, all patients were in complete remission of their blood diseases. Lymphopenia (less than 1500/mm^3^) was observed in six patients (14%), and hypogammaglobulinaemia defined by immunoglobulin electrophoresis was found in ten patients (23%). None of the patients had neutropenia. Seventeen patients (40%) were on immunosuppressive treatment at the time of vaccination (ciclosporin, *n* = 16; mycophenolatemofetil, *n* = 3; and methotrexate, *n* = 1). Six patients (13%) were on corticosteroid therapy.

The control group consisted of 31 healthy volunteers who received their second dose of the BNT162b2 vaccine between August 2021 and April 2022. They were randomly chosen from the Evax database. The median age was 50 years (40–53), significantly higher than that of patients (*p* < 0.001). The sex ratio was 0.82 (14 men, 17 women) (*p* = 0.366 when compared to patients). Eight controls had a previous symptomatic COVID-19 infection. The confirmation of infection was performed using RT-PCR (*n* = 7) and suggestive thoracic computed tomography with negative RT-PCR (*n* = 1). Among these controls, one had a severe form; three had a moderate form; and the last four had mild forms. The evolution was favorable for all the controls.

The characteristics of patients and controls are summarized in Table 1.

### 3.2. Clinical Tolerance of Vaccination

**Figure 2:**
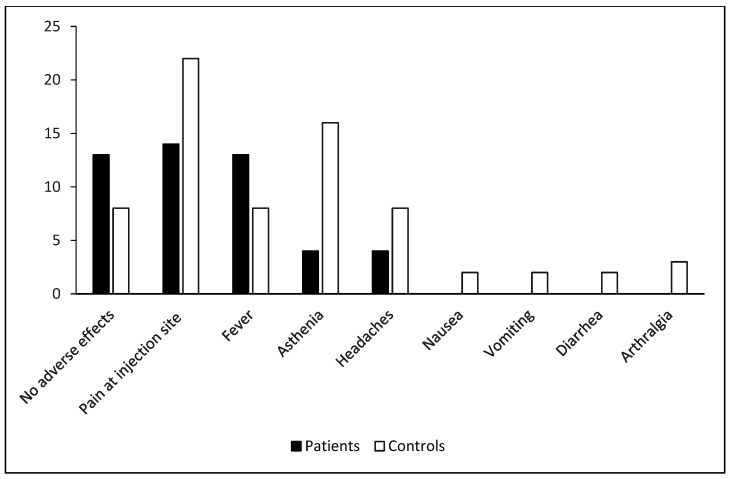
Adverse effects after anti-SARS-CoV-2 vaccination. The frequency of adverse effects after two doses of BNT162b2 is reported in 43 ASCT patients and 31 healthy controls.

More than half of the patients (*n* = 29) had mild-to-moderate adverse effects (grades 1–2) after anti-SARS-CoV-2 vaccination. Pain at the injection site was the most frequently encountered adverse effect (33%, *n* = 14), followed by fever (31%, *n* = 13), asthenia, and headache (10%, *n* = 4) (Figure 2). Several variables were analyzed in a univariate study as factors that could influence vaccine tolerance in ASCT patients: age, gender, the type of blood disease, anti-N antibody positivity, vaccination-transplant period, the presence of chronic active GVHD, and the current treatment (immunosuppressants and corticosteroids). In the univariate analysis, no factor had a significant impact on vaccine tolerance in ASCT.

Similarly, more than half of the controls (*n* = 23 or 74% of cases) had mild-to-moderate adverse effects (grade 1 or 2) after anti-SARS-CoV-2 vaccination. Pain at the injection site was the most frequently encountered adverse effect (*n* = 22 or 71% of cases), followed by asthenia (*n* = 16 or 52%) (Figure 2).

The variables analyzed in a univariate study as factors that could influence the occurrence of adverse effects in controls were age, gender, and anti-N antibody positivity. Only gender had a significant impact on vaccine tolerance in controls. Indeed, adverse effects in controls were significantly higher in females with a *p* = 0.007 (Table 2).

The comparative study of vaccination tolerance showed that the controls presented more adverse effects such as pain at the injection site and asthenia (*p* = 0.001, *p* < 0.001, respectively) than the patients, without any significant difference in the frequency of occurrence of adverse effects, all symptoms combined (*p* = 0.693).

### 3.3. Humoral Response after Vaccination

**Figure 3:**
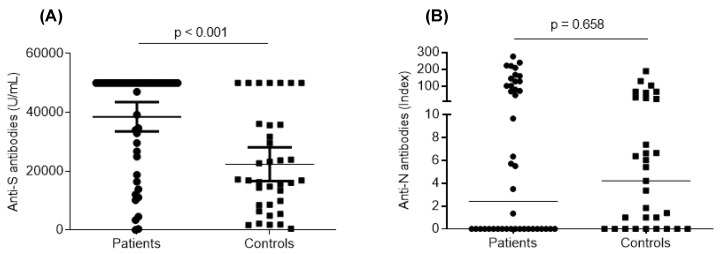
Humoral response after SARS-CoV-2 vaccination. Anti-S (**A**) and anti-N (**B**) antibody responses after SARS-CoV-2 vaccination were compared between ASCT patients (*n* = 43) and controls (*n* = 31). Along with dot plots, median values are shown. For comparison, the Mann–Whitney *U* test was used, and *p* values are shown.

The humoral response was assessed by evaluating SARS-CoV-2 anti-S RBD antibodies. All patients and controls developed anti-S antibody titers (>15.0 U/mL). Collectively, anti-S antibody titers were significantly higher in the ASCT patient group compared to the control group (*p* < 0.001) (Figure 3). In patient group, titers varied from 60.97 U/mL to 50,000 U/mL, with a median of 50,000 U/mL. In control group, titers varied from 20.64 U/mL to 50,000 U/mL, with a median of 16,078 U/mL. Based on a threshold of 1700 U/mL, considered protective according to Dimeglio et al. [8], 88% of patients (*n* = 38) and 90% of controls (*n* = 28) had protective titers. Interestingly, 28 patients (65%) and 4 controls (13%) had titers above 50,000 U/mL.

Regarding anti-N antibodies, 52.4% of patients (*n* = 22) were positive versus 61.3% of controls (*n* = 19) (*p* = 0.448)**.** The comparative study of anti-N antibody levels did not show any significant difference between patients and controls (*p* = 0.658) (Figure 3). Patients and controls with anti-N positivity included all the subjects with a biologically or radiologically documented history of COVID-19. For the rest of individuals, the positivity of the anti-N antibodies provided information on a history of COVID-19 that went unnoticed.

**Figure 4:**
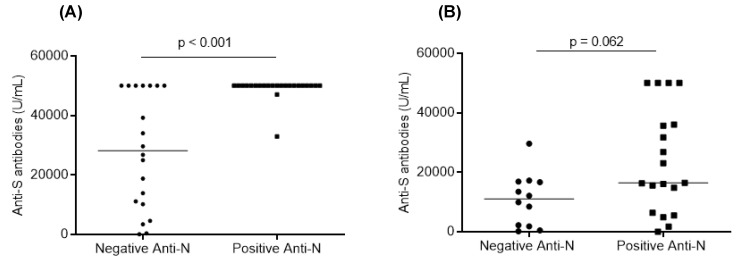
Distribution of anti-S antibody levels according to anti-N antibody positivity in patients (**A**) and controls (**B**). Along with dot plots, median values are shown. Mann–Whitney U test was used, and *p* values are shown.

The study of the positivity of anti-N antibodies as a factor that could influence the humoral response showed that this had a significant impact on patients (*p* < 0.001). In addition, patients with positive anti-N antibodies had significantly higher levels of anti-S antibodies (*p* < 0.0001). In fact, the median level of anti-S antibodies in patients with positive anti-N antibodies (50,000 U/mL) was significantly higher than that (28,180 U/mL) of patients with negative anti-N antibodies (Figure 4). Although the positivity of anti-N antibodies did not seem to significantly influence the humoral response in controls, the median level of anti-S antibodies was higher in controls with positive anti-N antibodies (16,472 U/mL versus 11,020 U/mL) (*p* = 0.062) (Figure 4).

### 3.4. Cellular Response after Vaccination

**Figure 5:**
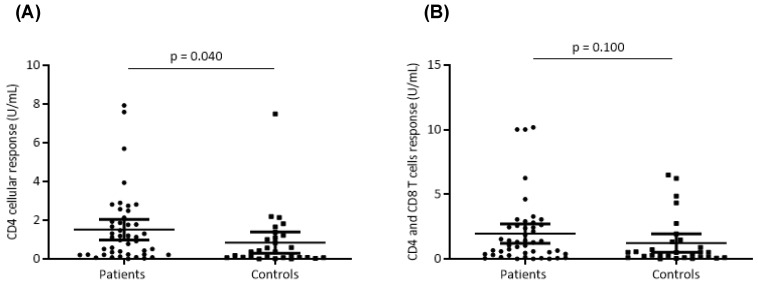
T cell response after SARS-CoV-2 vaccination. CD4^+^ (**A**) and CD4^+^ + CD8^+^ (**B**) T cell responses after SARS-CoV-2 vaccination were compared between patients and controls. Along with dot plots, median values are shown. Mann–Whitney U test was used, and *p* values are shown.

**Figure 6:**
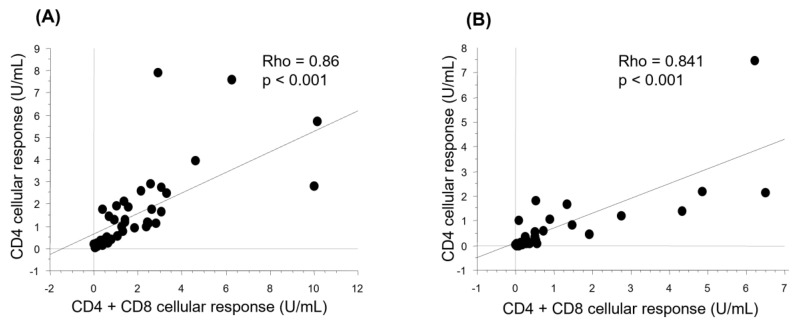
Correlation between CD4^+^ and CD4^+^ + CD8^+^ T cell responses. The correlation between the two types of cellular immune responses was analyzed using Spearman test in 43 patients (**A**) and 31 controls (**B**).

The cellular response was evaluated by measuring the levels of IFN-γ in the supernatants of QFN SARS-CoV-2 tubes. The positivity threshold was set at 0.15 U/mL by the supplier. A positive cellular response was found in 86% of patients (*n* = 37) versus 71% of controls (*n* = 22) (*p*= 0.176). The median IFN-γ level obtained with CD4 (Ag1) tubes was 1.15 U/mL (0.32–2.22 U/mL) in patients versus 0.36 U/mL (0.07–1.15 U/mL) in controls (*p* = 0.040) (Figure 5). This rate was 1.34 U/mL (0.32–2.22 U/mL) with the CD4 and CD8 (Ag2) tubes versus 0.49 U/mL (0.11–1.39) in the controls (*p* = 0.100) (Figure 5). Interestingly, CD4^+^ and CD4 ^+^ + CD8^+^ cellular responses were highly correlated both in patients (*p* < 0.001 and Rho = 0.86) and in controls (*p* < 0.001, Rho = 0.841) (Figure 6).

However, no correlation was found between the anti-S antibody levels and the cellular response, either for the patients or for the controls (Supplemental Figure S1). Strikingly, anti-N antibodies had no significant impact on the cellular response in both groups (Table 3 and Table 4).

### 3.5. Factors Associated with the Humoral and Cellular Responses after Vaccination

Several variables were analyzed in a univariate study as factors that could influence the immune response in ACST patients. The results are summarized in Table 3.

In ASCT patients, the delay between ASCT and vaccination had a significant impact on the post-vaccination humoral response (*p* = 0.004). The longer this delay, the higher the titer of anti-S antibodies. In fact, patients vaccinated after more than 12 months of transplantation had significantly higher levels of anti-S antibodies than those vaccinated during the first year post-transplant (*p* = 0.034). The same is true for patients vaccinated after more than 24 months of transplantation, who had significantly higher levels of anti-S antibodies than patients vaccinated during the first two years post-transplant (*p* = 0.018). This was not found with the cellular response. No significant difference between the CD4^+^ and CD4^+^ and CD8^+^ responses of patients vaccinated during the first year post-transplant and those vaccinated afterwards (*p* = 0.942, *p* = 0.673, respectively) was observed. The same is true for patients vaccinated during the first two years post-transplant and those vaccinated afterwards (*p* = 0.818, *p* = 0.556, respectively). Furthermore, the presence of chronic active GVHD had a significant positive impact on the post-vaccination CD4^+^ (*p* = 0.003) and CD4^+^ + CD8^+^ (*p* = 0.053) cellular responses.

As shown in Table 4, no factor had a significant impact on the post-vaccination humoral and cellular responses in control group.

By carrying out a multivariate analysis of the parameters that can influence the response and adjusting for age as a probable confounding factor, the only factor determining the humoral response remains the positivity of the anti-N antibodies.

Significantly, upon pooling both groups (patients and controls) and conducting a repeated univariate study, we observed a negative correlation between anti-S antibodies and age (*p* = 0.005). Moreover, the positivity of anti-N antibodies continued to exhibit a significant association with the humoral response (Table 5).

### 3.6. Cytokine Profile after Vaccination on a Sample of Patients and Controls

The analysis of the anti-SARS-CoV-2 cellular response was completed by the multiplex analysis of several different cytokines (IL-2, IL-4, IL-5, IL-6, IL-9, IL-10, IL-12,IL-17A, IL-17F, IL-22, IFN-γ, and TNF-α) in the supernatant of lymphocytes stimulated with SARS-CoV-2 antigens from QFN tubes in a subgroup of 10 ASCT patients and 10 controls. The choice of these subgroups of patients and controls was based on the representativeness of different levels of cellular response.

**Figure 7:**
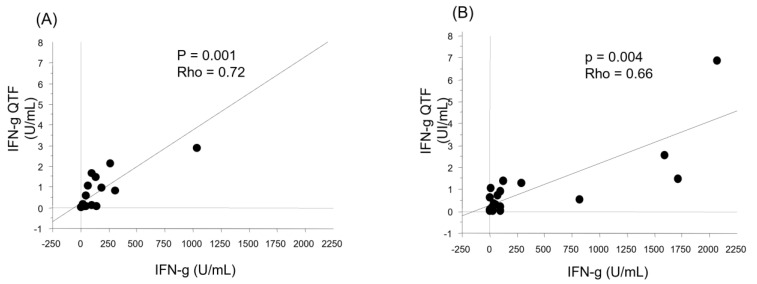
Correlation between the levels of IFN-γ determined by two different techniques. The correlation between the levels of IFN-γ determined with ELISA (IFN g QTF), and those estimated by the multiplex technique were analyzed in CD4^+^ (**A**) and CD4^+^ + CD8^+^ (**B**) samples.

As shown in Figure 7, the IFN-γ level quantified by the multiplex technique in the supernatants of the QFN CD4^+^ and CD4^+^ + CD8^+^ tubes was correlated with that assayed by the ELISA technique (*p* = 0.001, Rho = 0.72; *p* = 0.004, Rho = 0.66, respectively).

**Figure 8:**
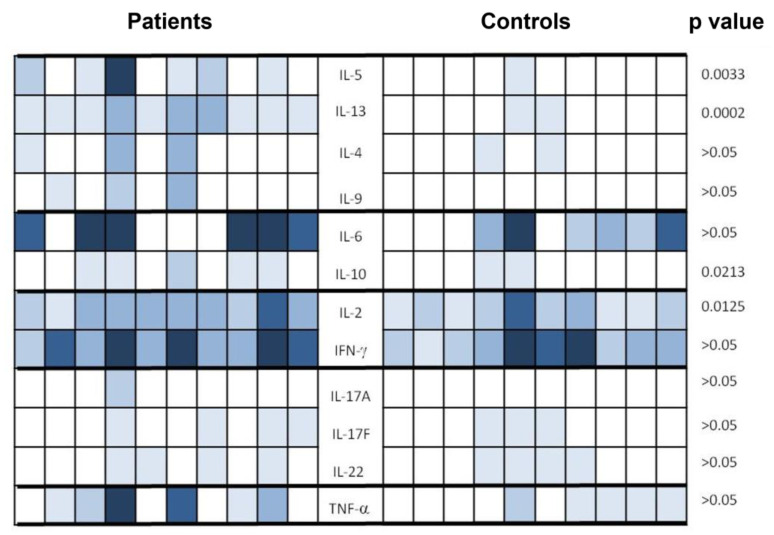
Schematic representation of Th1/Th2/Th17 cytokine profile in patients and controls. Th1, Th2, and Th17 cytokines were measured in the supernatant of lymphocytes stimulated with SARS-CoV-2 antigens from Quantiferon tubes in a subgroup of 10 ASCT patients and 10 controls. The superior result obtained within the CD4^+^ and the CD4^+^ and CD8^+^ tubes is shown. The intensity of the color is correlated with the cytokine level. Comparison of cytokine levels between patients and controls was performed using Mann–Whitney U test, and *p* values corresponding to each cy tokine are shown on the same line.

**Figure 9:**
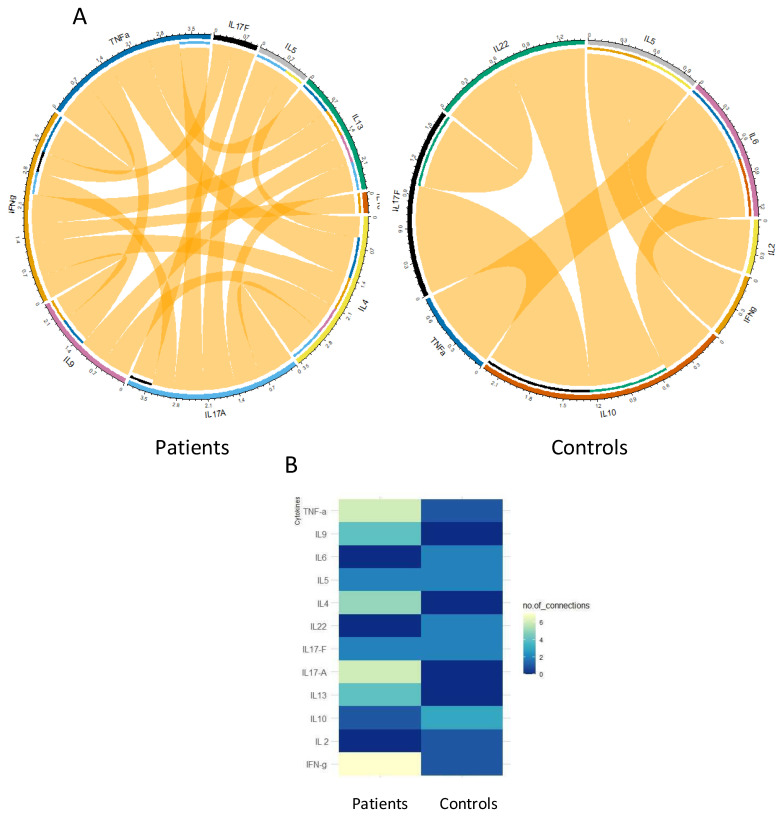
Network analysis of Th1/Th2/Th17 cytokine correlation matrices in patients and controls. (**A**) Circos plots illustrate the correlation networks built according to Spearman correlation matrices. Each bar represents a different parameter. The length of each bar is proportional to the number of significant correlations. The connecting lines represent statistically significant correlations (*p* < 0.05). Yellow connecting lines represent positive correlations. The thickness of the connecting lines is proportional to the Spearman correlation coefficient value. Markers that did not exhibit statistically significant correlations are now shown in the Circos plots. (**B**) Node analysis: heatmap shows the number of statistically significant correlations involving each marker per clinical group.

Figure 8 summarizes the results obtained by the multiplex technique. In this figure, the superior result obtained in the CD4^+^ and the CD4^+^ and CD8^+^ tubes is analyzed. Th1 cytokines (IL-2 and IFN-γ) are the most prominent cytokines detected in both groups with a difference that was significant for IL-2 in patients versus controls (*p* = 0.0213). Th17 interleukins (IL-17A, IL-17F, and IL22) were very weakly detected either in patients or in controls. Their rate was comparable between both groups. The level of the pro-inflammatory cytokine TNF-α was higher in the patients, but the difference was not significant (*p* > 0.05). The level of Th2 type cytokines was globally higher in patients compared to controls, particularly for IL-5 and IL-13 for which the difference was highly significant (*p* = 0.0033 and *p* = 0.0002, respectively). Finally, the median level of IL-10 was significantly higher in patients compared to controls (*p* = 0.0213). As shown in Figure 9, the correlation between the tested cytokines differs considerably between patients and controls, revealing higher connections in patients and reinforcing the distinction in cytokine profiles.

### 3.7. Clinical and Immunological Follow-Up

All patients were followed-up clinically for a period ranging from 4 to 11 months. During this period, ten patients developed SARS-CoV-2 infections, and of which six went unnoticed (positive anti-N antibodies despite initially testing negative) and four patients presented mild COVID-19 symptoms. Only 8 out of the 43 patients agreed to receive a third dose after 6 months, according to the vaccination schedule recommended by the Francophone Society for Bone Marrow Transplantation and Cellular Therapy (SFGM-TC).

All patients were invited for an immunological follow-up of the vaccine response, but only fifteen patients, of which five who received a third dose, participated in the follow-up. Among these 15 patients (Table 6), 6 had positive anti-N antibodies at the first sampling (after the second dose), and twelve had positive antibodies at the second sampling after follow-up. Regarding the anti-S responses, all patients still had positive anti-S antibodies, although this response decreased by more than half in seven patients. It was strengthened in two patients (P5 and P8) who had an asymptomatic SARS-CoV-2 infection during follow-up (the positivity of the anti-N antibodies during follow-up).

One patient (P14; 41 years old with acute leukemia) who initially had a positive humoral response and a negative cellular response, maintained the same humoral response profile with a positive level of anti-S antibodies (>2500 U/mL) and a negative QFN SARS-CoV-2 test, despite receiving a third dose of the Pfizer-BionTech^®^ vaccine. Patient P6 (20 years old with acute leukemia) who initially had a negative cellular response developed a cellular response after a breakthrough of SARS-CoV-2 infection. In fact, he developed anti-N antibodies (previously negative), indicating a past asymptomatic SARS-CoV-2 infection. Two other patients (P3 and P7) who initially had a good humoral and cellular responses maintained a humoral response with anti-S antibody levels lower than those measured after the second dose but still above the positivity threshold, but their cellular response became negative. These patients did not receive a third dose.

## 4. Discussion

Allogeneic hematopoietic stem cell transplantation (ASCT) is an essential therapy for several hematological diseases. However, it is burdened with significant morbidity and mortality partly linked to bacterial, viral, or fungal infections, occurring in a context of secondary immune deficiency. Indeed, the ASCT induces a profound and prolonged immune deficiency due to the systematic use of immunosuppressive treatments and the more or less long delay in immune reconstitution. As a result, HSC transplant recipients constitute a group particularly vulnerable to COVID-19. They present an increased risk of serious forms and mortality (nearly 20% against 2% in the general population). The management of patients with hemopathies and particularly HSC transplant patients was largely disorganized at the time of the epidemic peaks of SARS-CoV-2 infection. In order to protect these patients from the risk of contamination, postponements have been imposed in the therapeutic schedule (delays in chemotherapy treatments, transplant dates, follow-up appointments, etc.), leading to a risk of treatment failure, disease progression, and a risk of excess mortality.

Anti-SARS-CoV-2 vaccination is a priority in the vaccination schedule after ASCT. The vaccine response deserves to be studied in these patients, given the particularity of their immune status. Indeed, recent publications reported conflicting results regarding the quality of anti-SARS-CoV-2 vaccine response in ASCT patients compared to that in the general population [7]. The objective of our study was to evaluate the clinical tolerance and immunogenicity of anti-SARS-CoV-2 vaccination in ASCT. We set secondary objectives to study the factors influencing the vaccine response, to evaluate the duration of the protection conferred by the vaccine in this population by studying the kinetics of the vaccine response, and to establish the cytokine profile of the vaccine immune response.

In our study, 43 patients had received a complete anti-SARS-CoV-2 vaccination (two doses of the Pfizer-BioNTech^®^ vaccine in accordance with the recommendations of the treating physicians), i.e., nearly half of the eligible subjects. The control group included 31 healthy individuals randomly selected via the Evax platform, from all vaccinated during the same period, aged over 15 years. Anti-SARS-CoV-2 vaccination was generally well tolerated by patients and controls. More than half of patients (69%) had mild-to-moderate adverse effects (grades 1–2) after anti-SARS-CoV-2 vaccination versus 73% of controls. No severe adverse effects (grades ≥ 3) requiring hospital treatment were noted. Our results seem to be consistent with data from the literature. In a single-center prospective cohort study, conducted by Ram et al., Pfizer-BioNTech^®^ vaccine-related side effects occurred in 39% of ASCT patients (*n* = 65). The most common side effects were the worsening of cytopenias (12%) and headaches (6%) [9]. Similar results were observed in the study by Ali et al., involving 113 ASCT patients who received the Pfizer-BioNTech^®^ or Moderna^®^ vaccines. Only 48 patients were evaluated for safety after vaccination, and the adverse effects occurring in 45% of them were mainly pain at the injection site (43.8%), followed by asthenia (29.2%) and myalgia–arthralgia (14.6%) [10]. Similar to our results, Ali et al. observed fewer adverse effects in ASCT patients than in controls. This could be explained by the presence of an active GVHD in 46% patients treated with corticosteroids that could mask the inflammatory phenomena responsible for certain adverse effects.

In our study, all patients developed antibodies titers considered neutralizing (>15.0 U/mL), ranging from 60 U/mL to 50,000 U/mL. Based on the threshold of 1700 U/mL, considered protective against infection according to Dimeglio et al. [8], 88% of patients were responders versus 90% of controls (*p* > 0.05). These results are consistent with those reported by other studies [9,11,12,13,14], which reported a humoral response rate between 66% and 83% in ASCT patients who received mRNA vaccination. However, other studies reported lower quality humoral responses in ASCT patients. In the study by Chiarrucci et al., which involved 50 transplanted patients (autograft, *n* = 38; allograft, *n* = 12), the administration of the Pfizer-BioNTech^®^ vaccine was capable of generating a humoral immune response in 84% of autograft patients but only in 50% of ASCT patients [15]. Chevallier et al. [16] studied the humoral response after the first dose of the Pfizer-BioNTech^®^ type vaccine in 112 ASCT patients. Only 55% of the patients had developed anti-S antibodies versus 100% of the control group with a significant *p* (*p* < 0.001). It should be noted that the median age of the patients included in this study was 57 years (20–75), which may explain the altered humoral response compared to those of the patients included in our series, who are much younger (median age: 30 years). This conclusion is also underlined in the study by Mamez et al. [17] that compared the results of its cohort made up of 63 ASCT patients that had received a vaccination of the Pfizer-BioNTech^®^ or Moderna^®^ type with other studies evaluating the humoral response after anti-SARS-CoV-2 vaccination in the general population with the same vaccines. The percentage of humoral response was lower (76%) in the ASCT group versus 94% in the general population.

In our study, only one factor had a significant impact on the post-vaccination humoral response in the univariate analysis: the duration between transplant and vaccination (*p* = 0.004). The longer is the delay, the higher the titer of anti-S antibodies. Similar results were observed in the study by Mamez et al. [17]. Indeed, the humoral response based on the dosage of anti-S antibodies after the first dose of vaccine was significantly lower when the post-transplant vaccination delay was less than 6 months (46%) versus 84% for a delay of more than 6 months. Redjoul et al. [12] also reported a negative impact on the vaccine response to vaccination during the first year post-transplant. This significant parameter in the univariate analysis has not been validated in the multivariate analysis. Another study conducted by Chevallier et al. found that the humoral response of ASCT patients was similar to that of the control group only in ASCT patients for more than 2 years [16]. Finally, in the recent study by Henig et al. [14], the humoral response rates for ASCT patients vaccinated at later (>12 months), intermediate (between 6 and 12 months), or early (between 3 and 6 months) time points were 83.7%, 52.4%, and 30.7%, respectively. Notably, several other factors impacting the humoral response have been identified in the literature. According to the study conducted by Huang et al. on 114 ASCT patients whose median age was 57 years (18–74), the rate of anti-S antibodies was significantly higher in patients receiving vaccination 12 months after receiving an allograft. However, age over 65 and the use of immunosuppressants were also associated with lower levels of antibodies [18]. These results were also consistent with those of Chiarucci et al. [15], which concluded that an altered humoral response was associated with an anti-CD20 therapy and the use of immunosuppressants or corticosteroids. The study by Bourgeois et al. [11] identified other factors associated with a poor humoral response in addition to the delay between ASCT and vaccination (<1 year): the administration of an immunosuppressive treatment at the time of vaccination, lymphopenia < 1 G/L, and the haploid transplant. Finally, our study did not note any statistically significant association between gender and post-vaccination humoral response, unlike results of the study by Lindemann et al. [19]. One of the limitations of our study is the analysis of the immunogenicity of only one vaccine, i.e., Pfizer-BioNTech^®^, for homogenizing the cohort. It would still be interesting to compare the impact of the type of the used vaccine on the post-vaccination immune response as conducted by others [17,20,21].

In immunocompromised patients in general and in ASCT patients in particular, most published studies have focused on the humoral component of post-vaccination immune responses against SARS-CoV-2 [11,12,15,16,18,20]. However, the cellular immune response in particular that involves IFN-γ-producing CD4^+^ and CD8^+^ T cells is crucial for fighting viral infections. T cell responses are associated with a lower risk of developing disease following a SARS-CoV-2 infection [22] and protection against the severe forms of COVID-19 [23,24,25]. Moreover, such a response is more durable than the humoral response [26] and less susceptible to variants of SARS-CoV-2, exhibiting humoral immune escape phenotypes [27,28]. The published studies of cellular immunity in ASCT have reported variable results, probably due to the different performances of the used techniques. In our study, the cellular response was assessed by measuring the level of IFN-γ using an interferon gamma release assay (IGRA) test (Quantiferon SARS-CoV-2 (Qiagen^®^)) whose effectiveness has been widely proven in SARS-CoV-2 infection [29,30,31,32]. Our data showed that 86% of our ASCT patients developed a positive cellular immune response versus 71% of controls. CD4^+^ and CD8^+^ T cell responses were strongly correlated. In a study by Gavriilaki et al. [33], which included 31 ASCT patients and 5 autograft patients and in whom the post-vaccination cellular immune response was studied using the ELISpot technique, a positive cellular response was found in 79% patient after the second vaccine dose. In studies by Lindeman et al. [19] and Clémenceau et al. [34], a shorter period between the transplant and vaccination predicted a poorer cellular response. This was not shown in our study. In fact, only the presence of an active GVHD had a significant positive impact on the cellular response in our cohort. This seems inconsistent with the results reported by Jimenez et al., in which active GVHD was rather associated with a poor cellular response [35]. This result should be verified on a larger sample.

Regarding the anti-N antibody analysis, this was performed to differentiate the immunity induced by infection from that induced by vaccination [8]. In our study, 52% of patients were positive versus 61% of controls. This positivity rate is relatively higher than that found in the general Tunisian population (38%) in April 2021 [36] and lower than that found in certain populations such as the Orthodox population in the United States (70%) (study carried out between May and July 2020) [37] but much higher than in other populations (27% in the Swiss population, and study carried out between June and July 2021) [38]. This could be explained by the inevitable increase in seroprevalence during a pandemic and the difference in health measures employed in different countries. Indeed, our study was carried out during the period corresponding to the fifth wave of COVID-19 in Tunisia (September–October 2021), and unfortunately, precise figures for the seroprevalence of COVID-19 in Tunisia during this period are not available.

In healthy immunocompetent subjects, anti-SARS-CoV-2 vaccination triggers a rather polarized Th1 cellular immune response with the production of IL-12 and IFN-γ and the activation of CD4^+^ T lymphocytes in 100% of patients and cytotoxic CD8^+^ cells in 92% of cases [39]. Our results are consistent with these data. Indeed, the patients and controls included in our study produced significant levels of IFN-γ with no significant difference between both groups. As expected, IL-2 levels were correlated with IFN-γ levels, even though they were significantly higher in patients compared to controls. The high level of IL-2 in the patients cannot be explained by the particular contexts of the patients (ASCT and GVHD) since studies have proven that the levels of IL-2 were low to undetectable in this group of patients [40]. The difference could be attributed to the age difference between the groups. The immune response to vaccination was worse in the elderly [41,42,43]. One of the most striking results of this study is that the levels of Th2 cytokines were more frequently detectable in patients at significantly higher levels. The profile of the cellular immune response induced in patients is thus mixed and is therefore less suited to an antiviral response. Nevertheless, this could partially elucidate the higher antibody levels observed in ASCT patients, as Th2 cytokines, particularly IL-4 and IL-13, are recognized as the potent stimulators of antibody production. Our patients had significantly higher IL-10 concentrations than controls. This could be a part of the observed Th2 profile, with IL-10 being a Th2 cytokine [44]. This increase could also denote an anti-inflammatory response that takes place each time there is an inflammatory phenomenon [45]. Moreover, our data show an increase in the inflammatory cytokine TNF-α in patients compared to that in controls, without any significant difference between them. Th17 interleukins were very weakly detected in patients and controls, indicating the absence of Th17 immune responses that could exert a deleterious effect during a viral [46] or inflammatory [47,48] response. A study on a larger cohort would make it possible to draw more robust conclusions on the cytokine profile of the post-vaccination response. Particularly, the mechanisms underlying the skewing towards a Th1/Th2 profile in ASCT patients should be addressed.

Our study has several strengths. This study focused on a global health priority, namely, the anti-SARS-CoV-2 vaccination. In addition, the included population (ASCT patients) presents interesting particularities to study in relation to the post-transplant immune status and its impact on the vaccine response. The study of vaccine tolerance and efficacy is of major interest in this population which was excluded from phase III therapeutic trials of anti-SARS-CoV-2 vaccines. From a methodological point of view, this is a prospective controlled study. The vaccine response in the ASCT group was compared to that of a group of healthy controls. Moreover, our study focused on both the humoral and cellular aspects of the post-vaccination response in ASCT subjects and is the first study to study the cytokine profile of the induced response. However, our study has some limitations. The study of the immune response was not carried out after the first dose of the vaccine due to the difficulties in moving patients living throughout Tunisia during a period of confinement and the risks of SARS-CoV-2 infection posed by these displacements for this vulnerable population. The small size of the cohort hampered the analysis of the factors associated with the vaccine response, even if our study collected all the ASCT subjects of the CNGMO, the only ASCT center in Tunisia, during the designated period of the study. Regarding the control group, the subjects included were randomly chosen did not match the patients from the age point of view. Consistently, the median age was 50 years for the controls against 34 years for the ASCT patients, with the possible implications on the vaccine response, which seems to deteriorate with age [41]. During the study period, the vaccination campaign prioritized elderly subjects, making it impossible to perfectly match controls with patients according to age. The subsequent recruitment of younger controls posed the risk of collecting individuals with a history of infection with other variants of SARS-CoV-2, a source of new biases.

Altogether, our results demonstrate an excellent immune response induced after two doses of the SARS-CoV-2 mRNA vaccine and are not in favor of a schedule with three doses of vaccines in primary vaccination as proposed by scientific societies (HAS and FDA). Several factors that may explain the good response observed in our cohort such as the young age of our ASCT patients (the eligibility for ASCT after myeloablative conditioning reserved for patients of <50 years), high positive anti-N antibody levels in the general Tunisian population and ASCT patients (equivalent to an immune boost), and the duration between the allograft and the vaccination exceeded 12 months in most patients (time allowed the restoration of immune competence). Moreover, more than half of our patients were not taking immunosuppressive therapy at the time of vaccination. However, it seems crucial to enlarge our cohort and to follow-up all vaccinated ASCT patients during 12 to 18 months in order to assess the persistence of the immune response and to monitor the occurrence of adverse effects in the medium term.

## 5. Conclusions

Our data indicate that Pfizer-BioNTech^®^ anti-SARS-CoV-2 vaccination is well tolerated in ASCT patients, inducing robust humoral and cellular responses. This raises questions about the necessity of a third dose in the initial ACST vaccination schedule. Additionally, our results demonstrate the presence of a Th1/Th2-type cytokine profile in ASCT patients. Further exploration is warranted to understand the impact of such a cytokine profile.

## Figures and Tables

**Table 1:** Characteristics of patients and controls.

	Patients (*n* = 43)	Healthy Controls (*n* = 31)
Age (Range)	31 (17–47)	50 (40–53)
Male	24	14
Female	19	17
History of COVID infection	4	8
Median duration from transplant to vaccination (months) (Range)	28 (3–54)	NA
Underlying disease	31 AL 6 AA 2 MDS 2 HL 2 CML	NA
Peripheral stem cells graft	21	NA
Bone marrow graft	22	NA
Lymphopenia	6	NA
Hypogammaglobulinemia	10	NA
History of acute GVHD	15	NA
Active controlled chronic GVHD	21	NA
Ongoing immunosuppressive treatment	17	NA
Long term corticosteroids	6	NA

AL: acute leukemia; AA: aplastic anemia; MDS: myodysplastic syndrome; HL: Hodgkin lymphoma; CML: chronic myeloid leukemia; and NA: not applicable.

**Table 2:** Univariate study of factors associated or correlated with side effects after SARS-CoV-2 vaccination in ASCT patients and controls.

Variable	Correlation/Association with Side Effects in Patients (*p*=)	Correlation/Association with Side Effects in Controls (*p*=)
Age	0.345	0.651
Gender	0.748	0.007
Duration from ASCT to vaccination	0.252	NA
AA versus MH	0.466	NA
History of acute GVHD	0.429	NA
Active chronic GVHD	0.675	NA
IS within 3 months	0.729	NA
Long-term steroid therapy	1.000	NA

AA: aplastic anemia; MH: malignant hemopathy; IS: immunosuppressive drugs; and NA: not applicable.

**Table 3:** Univariate study of factors associated or correlated with the humoral and cellular responses after SARS-CoV-2 vaccination in ASCT patients.

Variable	Correlation/Association with Humoral Response (*p*=)	Correlation/Association with CD4^+^ Cellular Response (*p*=)	Correlation/Association with CD4^+^ + CD8^+^ Cellular Response (*p*=)
Age	0.560 (Rho = −0.090)	0.807 (Rho = 0.039)	0.738 (Rho = 0.053)
Gender	0.405	0.870	0.536
Side effects after BNT162b2 vaccination	0.619	0.686	0.523
Duration from ASCT to vaccination	0.004 (Rho = 0.434)	0.581 (Rho = 0.088)	0.368 (Rho = 0.142)
AA versus MH	0.774	0.652	0.631
Absolute lymphocyte count (<1 × 10^9^/L)	0.388 (Rho = 0.137)	0.356 (Rho = −0.183)	0.252 (Rho = −0.170)
Hypogammaglobulinemia	0.175	0.566	0.501
History of acute GVHD	0.683	0.157	0.467
Active chronic GVHD	0.926	0.003	0.053
IS within 3 months	0.072	0.812	0.944
Long-term steroid therapy	0.107	0.102	0.115
Anti-N antibodies (+)	<0.001	0.467	0.476

IS: immunosuppressive drugs other than steroids; AA: aplastic anemia; and MH: malignant hemopathy.

**Table 4:** Univariate study of factors associated or correlated with the humoral and cellular responses after SARS-CoV-2 vaccination in controls.

Variable	Correlation/Association with Humoral Response (*p*=)	Correlation/Association with CD4^+^ Cellular Response (*p*=)	Correlation/Association with CD4^+^ + CD8^+^ Cellular Response (*p*=)
Age	0.070 (Rho = −0.330)	0.096 (Rho = 0.315)	0.710 (Rho = 0.072)
Sex	0.068	0.121	0.597
Anti-N antibodies (+)	0.062	0.185	0.157

**Table 5:** Univariate study of factors associated or correlated with the humoral and cellular responses after SARS-CoV2 vaccination, considering both patients and controls in a pooled analysis.

Variable	Correlation/Association with Humoral Response (*p*=)	Correlation/Association with CD4^+^ Cellular Response (*p*=)	Correlation/Association with CD4^+^ + CD8^+^ Cellular Response (*p*=)
Age	0.005 (Rho = −0.425)	0.241 (Rho = −0.141)	0.344 (Rho = −0.114)
Sex	0.960	0.545	0.792
Anti-N antibodies (+)	<0.001	0.355	0.457

**Table 6:** Post-vaccine immune response follow-up in ASCT patients.

Vaccine Immune Response at the First Sampling *						Vaccine Immune Response at the Second Sampling					Follow up Duration (Months)	3rd Dose 1 = Yes/0 = No
Humoral Response			Cellular Response			Humoral Response		Cellular Response				
anti-N		anti-S	CD4	CD4 CD8	QFN Result	anti-N	anti-S	CD4	CD4 CD8	QFN Result		
P1	145.3	50,000	0.970	1.278	1	182.7	12,500	2.682	2.799	1	6	1
P2	72.4	50,000	1.125	2.462	1	32.2	13,100	0.230	0.860	1	11	0
P3	0	50,000	2.813	10.024	1	67.5	43,580	0.084	0.058	0	11	0
P4	220.6	50,000	1.764	2.636	1	247.4	50,000	3.095	3.245	1	8	0
P5	0	3434	5.698	10.171	1	202.8	40,480	6.755	3.585	1	5	1
P6	0	50,000	0.087	0.132	0	14.59	50,000	0.144	0.302	1	10	0
P7	68.86	50,000	1.925	1.029	1	126.9	9675	0	0	0	10	0
P8	0	60.97	0.230	0.150	1	173.2	50,000	4.523	1.659	1	10	0
P9	209	50,000	1.112	2.804	1	25.2	50,000	3.262	3.443	1	10	0
P10	5.5	50,000	0.385	0.747	1	5.95	17,772	0.872	0.537	1	10	0
P11	0	50,000	3.938	4.612	1	78.44	50,000	1.275	1.309	1	11	0
P12	0	10,170	0.346	0.270	1	18.15	10,315	0.295	0.237	1	10	0
P13	0	50,000	1.865	1.528	1	0	12,500	0.440	0.390	1	4	1
P14	0	18,792	0.082	0.075	0	0	2500	0.010	0.013	0	5	1
P15	0	39,200	2.813	10.021	1	0	12,500	1.511	5.295	1	6	1

* two weeks after the second dose of anti-SARS-CoV-2 BNT162b2 mRNA vaccine.

## Data Availability

All data of this study are available in the main text.

## Supplementary Materials

The following supporting information can be downloaded at: https://www.mdpi.com/article/10.3390/vaccines12020174/s1, Figure S1: Correlation between anti-S antibody levels and cellular responses in patients and controls.
