# Supplementary material for: Immunogenicity and Tolerance of BNT162b2 mRNA Vaccine in Allogeneic Hematopoietic Stem Cell Transplant Patients
Source: Vaccines (Basel). 2024 Feb 8;12(2):174. doi: 10.3390/vaccines12020174 (PMC10892348; doi:10.3390/vaccines12020174)
Supplement: Supplementary file 1 [file vaccines-12-00174-s001.zip › vaccines-2822289-supplementary.pptx]

## Slide 1
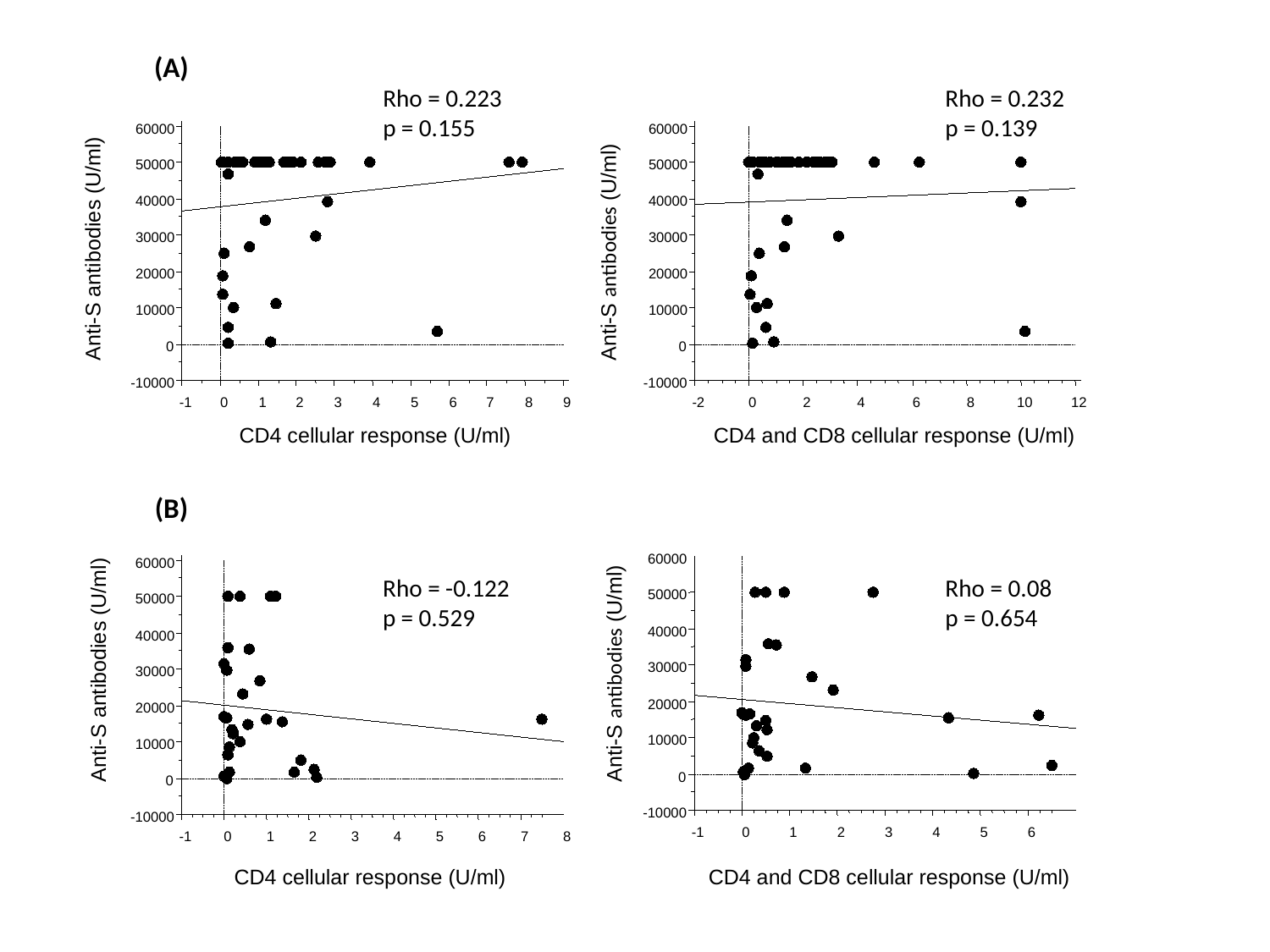

(A)
Rho = 0.223
p = 0.155
Rho = 0.232
p = 0.139
60000
60000
50000
50000
40000
40000
30000
30000
Anti-S antibodies (U/ml)
Anti-S antibodies (U/ml)
20000
20000
10000
10000
0
0
-10000
-10000
-1
0
1
2
3
4
5
6
7
8
9
-2
0
2
4
6
8
10
12
CD4 cellular response (U/ml)
CD4 and CD8 cellular response (U/ml)
(B)
60000
60000
Rho = -0.122
p = 0.529
Rho = 0.08
p = 0.654
50000
50000
40000
40000
Anti-S antibodies (U/ml)
30000
Anti-S antibodies (U/ml)
30000
20000
20000
10000
10000
0
0
-10000
-10000
-1
0
1
2
3
4
5
6
-1
0
1
2
3
4
5
6
7
8
CD4 cellular response (U/ml)
CD4 and CD8 cellular response (U/ml)
